# Hospitalization rates and resource utilization of schizophrenic patients switched from oral antipsychotics to aripiprazole-depot in Germany

**DOI:** 10.1186/s13561-018-0215-5

**Published:** 2018-11-23

**Authors:** Christoph Potempa, Reinhard Rychlik

**Affiliations:** Institute of Empirical Health Economics, Am Ziegelfeld 28, 51399 Burscheid, Germany

**Keywords:** Schizophrenia, Aripiprazole-depot, Long-acting injectable, Hospitalization, Budget-impact-analysis

## Abstract

**Objective:**

Examine cost-driving factors of schizophrenia in Germany for patients prior- and post-switch from an oral antipsychotic therapy to aripiprazole-depot and perform a budget impact analysis (BIA) referring to the context of German health care.

**Methods:**

A single-armed, retrospective, non-interventional pre-post comparison study with 132 patients to compare the total psychiatric hospitalization rates and the associated costs of both, the treatment with oral antipsychotics and aripiprazole-depot. The BIA was performed to compare both treatment periods with respect to health-related costs. A subsequent univariate sensitivity analysis examined the robustness of the results.

**Results:**

After switching the treatment to aripiprazole-depot, the total psychiatric hospitalization rates for the 6-month treatment period were significantly (*p* < 0.001) lower (14%) compared to the hospitalization rates when treated with oral antipsychotics (55.1%). 18.2% of the patients reported to be employed, with 29.2% having work incapacities. The mean number of schizophrenia episodes was 2.58 episodes per patient during the oral-antipsychotic treatment compared to 0.41 episodes per patient during the aripiprazole-depot phase (*p* < 0.001). The treatment with aripiprazole-depot also significantly reduced the mean number of hospitalizations per patient (0.63 to 0.16, *p* < 0.001) and the mean number of hospitalized days (27.39 to 5.56, *p* < 0.001) compared to the oral antipsychotic treatment. Additionally a significant reduction of the mean stay in day-clinics and psychiatric institute ambulances (PIAs) was observed (46.13 days to 7.29 days, *p* < 0.01).

Treatment of a patient suffering from schizophrenia with oral antipsychotics produced costs of 9935.38€ (direct costs: 9498.36 €), while aripiprazole-depot generated costs of 4557.56€ (direct costs: 4449.83 €) per patient for a one-year observation period. This resulted in total costs of 6,517,606,265.43€ for the oral antipsychotic treatment and 2,989,756,603.05€ for aripiprazole-depot treatment from the perspective of the German health care system. The results remained robust during sensitivity analysis, with aripiprazole-depot being the more cost-effective strategy.

**Conclusions:**

The results suggest that aripiprazole-depot treatment for schizophrenia patients has major potential in terms of cost savings for the German statutory health insurance.

## Introduction

Schizophrenia is estimated to affect 24 million individuals worldwide (656,000 - 738,000^1^ [1] in Germany considering a prevalence rate between 0.8% and 0.9% for patients between 18 and 65 years [2]). Total costs related to schizophrenia were estimated at $62.7 billion in the US [3], and the total costs of schizophrenia and other psychotic disorders were estimated at 93.9 billion € in Europe [4]. The annual direct costs of schizophrenia treatment per patient in Germany are estimated between 14,000-18,000€ [5] with hospitalizations and productivity losses as the main cost-driving factors. The annual total costs for schizophrenia in Germany are estimated between 4,400,000,000€ and 9,200,000,000€ [6].

The World Health Organization (WHO) estimates the costs of schizophrenia in western countries in a range between 1.6% and 2.6% of total health care expenditures. The social and economic consequences of mental illnesses are considerable high, with its treatment costs surpassing even heart diseases, cancer, and diabetes treatment [7].

The direct costs generated by schizophrenia comprise all resource consumption resulting from a treatment or therapy and are directly attributable to this disorder like medication costs, hospitalizations, outpatient and long-term care [8].

The indirect costs occur not directly in relation to the treatment of the disease but arise from the fact that schizophrenia is a very disabling disease. Schizophrenia is associated with productivity losses by the patient and the patient’s family members. Work incapacities (temporary or definitive) and disability pensions due to schizophrenia can be very cost-intensive for the health care system [9]. Less reliable data are available on the indirect costs because resource- and productivity losses as a result of morbidity and mortality in the context of schizophrenia are more difficult to access [7].

Another reason for the high costs of schizophrenia arises from the fact that it is a chronic and lifelong condition. The resource consumption of patients with schizophrenia in Germany has not been evaluated in detail. Due to the cost-intensive characteristics of schizophrenia a reduction of these costs could result in significant savings from the perspective of the German statutory health insurance.

The reduction of the direct and indirect costs of schizophrenia can be realized by different procedures. Better and more cost-effective medications can be recognized as one of those solutions. Sustained release drugs (like aripiprazole-depot) are reported to have significantly higher adherence rates than traditional oral antipsychotics [10].

The findings on the effectiveness of aripiprazole-depot are also consistent with several clinical studies that associate reduced psychiatric hospitalization rates and durations with sustained release drugs compared to traditional oral anti-psychotic therapies [11].

Recent meta-analyses came to the conclusion that sustained release drugs are able to reduce the hospitalization risk significantly compared to oral antipsychotic treatments [12, 13]. A total of 58 evaluated studies show that the reduction in hospitalization rates for LAIs (long-acting injectable) was 20.7 percentage points higher than that of OAs (oral antipsychotics) (LAIs = 56.2% vs. OAs = 35.5%, *P* = 0.023) [13].

The main objectives of this study can be summarized in two sub goals:

- Examine whether aripiprazole-depot treatment reduces the hospitalization rates and other cost driving factors compared to oral medication treatment for schizophrenia.
- Compare conceivable benefits and cost-savings (including direct and indirect costs) for the German statutory health insurance for both observation phases based on several variables of resource consumption over a one-year observation time frame.

## Methods

### Study design and observation group

This study was carried out as a single armed, non-interventional pre-post comparison study with a subsequent BIA to assess hospitalization rates in patients with schizophrenia treated with oral antipsychotics followed by a treatment with aripiprazole-depot in a community setting in Germany. The study consisted of two retrospectively reported treatment-phases. The retrospective documentation period started in May 2016 and had been finalized in November 2016.

**Fig. 1:**
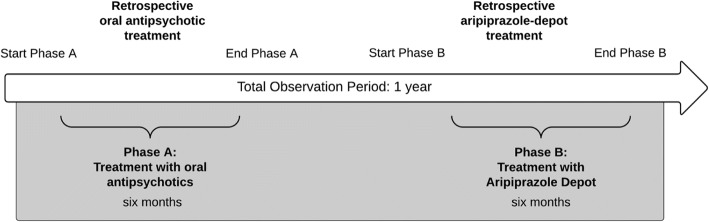
Study design and time frame

The required sample size was estimated at *n* = 116 to provide 80% power to detect a statistically significant difference of 11% between the hospitalization rate during the aripiprazole-depot treatment period (Phase B) and a recently published hospitalization rate for patients being treated with oral antipsychotics (38.1%) from the mentioned study [11]. A dropout rate of 10% was assumed, which resulted in a required total sample-size of *n* = 129 patients (Fig. 1).

A total of 6994 physicians were contacted. The total number of recruited centers (psychiatrists) was 67 (from which 28 (41.8%) were active with the 39 (58.2%) remaining centers counting as dropouts due to lack of transmitted patient data). Data for 136 patients was collected at the end of the inclusion phase by a total of 28 psychiatrists in Germany. The database was closed on November the 30th 2016. 4 of those 136 patients (2.9%) were excluded from further analysis because of not meeting the inclusion criteria (18–65 years) of the study.

Data was documented for two different phases. During the six-month observation period phase A the patients schizophrenia was treated with traditional oral antipsychotics. With the end of phase A the treatment was switched to aripiprazole-depot for six subsequent months. The observation periods both had a duration of six months each, regardless whether the treatment itself lasted longer than six months. This condition ensured comparability between both treatment phases. Variables from the following three categories were retrieved during data collection phase and were considered in the statistical analyses:

- Socio-demographic and administrative parameters
- Indication- and efficacy based parameters
- Parameters of resource-utilization

### Study eligibility

Eligible patients (*n* = 132) were aged between 18 and 65 years with a schizophrenia diagnosis consistent with the International Statistical Classification of Diseases and Related Health Problems (ICD-10). Patients also needed to have been prescribed an oral anti-psychotic treatment in the six months prior to the switch to aripiprazole-depot, which had to be documented for six subsequent months.

Eligible patients also required a change in treatment for any reason, with the specific reason being reported by the investigator (e.g. insufficient efficacy, insufficient compliance, side effects). This change in treatment represents no intervention (compared to RCTs) because of the retrospective character of the study. Eligibility was determined retrospectively by the investigator, which explains the low amount of patients that failed to meet the inclusion criteria of the study (4 patients).

Subjects with an ICD-10 diagnosis other than schizophrenia (2 patients with schizoaffective disorder) were included in the analysis because of being reported in multiple categories and having a schizophrenia diagnosis as well.

Some patients (the amount differed between variables) were excluded from several statistical procedures because of item-non-response or contradictions concerning the extent of some of the reported outcome variables. Hospitalized days for example or days with work incapacities were very occasionally reported for longer time-frames than the actual observation time-period (six months per phase).

### Endpoints

The primary endpoint was the psychiatric hospitalization rate (defined as the proportion of patients with ≥1 psychiatric hospitalization) which was assessed and compared between the oral anti-psychotic treatment period (months 1–6) and the aripiprazole-depot treatment period (months 7–12).

Additionally secondary efficacy and endpoint comparisons were compared between both treatment periods including the number of schizophrenia episodes, the mean duration of hospitalizations, the number of emergency treatments (emergency physicians and emergency services), the frequency and duration of day-clinic-visits as well as the number of psychiatrist visits and productivity losses in form of days of sick leave.

### Statistical analysis

Comparisons between hospitalization rates during the retrospective oral anti-psychotic treatment period (months 1–6) and the aripiprazole-depot treatment period (months 7–12) were assessed using Wilcoxon signed rank tests with a statistical significance at an alpha level of 0.05 (two-sided) after using Kolgomorov-Smirnov tests to check the observed empirical distributions for normality.

Additionally the hospitalization rate of patients during the treatment with aripiprazole-depot was compared with the hospitalization rates conducted by a recent study by Kane et al. [11] using a one-sided binomial test.

### Budget-impact-analysis

The BIA is a specific form of health economic evaluation, which evaluates the financial consequences of new technologies in a specific health care system [14]. The analysis form is increasingly required in national reimbursement procedures and can also be used for resource or budget planning [15].

In this investigation, a BIA was performed to estimate the annual budget impact of two different treatment options for schizophrenia. The analysis referred to the context of German health care, was performed from the perspective of the German statutory health insurance and considered direct and indirect costs of schizophrenia to deliver an overview on the resource utilization and the related costs of patients suffering from schizophrenia. It should be noted here that productivity losses in Germany only become relevant from the point of view of the statutory health insurances starting with the sixth week of sick leave. Therefore, the direct costs were evaluated separately in order to avoid misunderstandings regarding the direct and indirect costs of schizophrenia.

## Results

### Demographics

Demographics and baseline characteristics for the 132 patients that received treatment with aripiprazole-depot after the switch from oral antipsychotics are reported in Table 1. The majority of patients were female (*n* = 71/132, 53.8%) and the mean age was 37.89 (sd = 10.54) years with a mean age at schizophrenia diagnosis of 30.55 years (sd = 8.67). 92.4% of the patients were insured statutory (*n* = 122/132) while 72 of these patients reported to have a co-payment exemption.

**Table 1:** Demographics

Measure	
Age, mean (SD), years	37.81 (10.54)
Male/female, n	60/71
Body weight, mean (SD), kg	84.94 (19.61)
Body height, mean (SD), cm	171.05 (8.56)
BMI, mean (SD), kg/m^2^	28.96 (6.17)
Age at schizophrenia diagnosis (SD), years	30.55 (8.67)
Employed / unemployed / retired / education / NA, n	24 / 39 / 51/ 9 / 9
Single / married / widowed / other /NA, n	80 / 43 / 3 / 5 / 1

### Primary efficacy end-point: Total psychiatric hospitalization rates

**Fig. 2:**
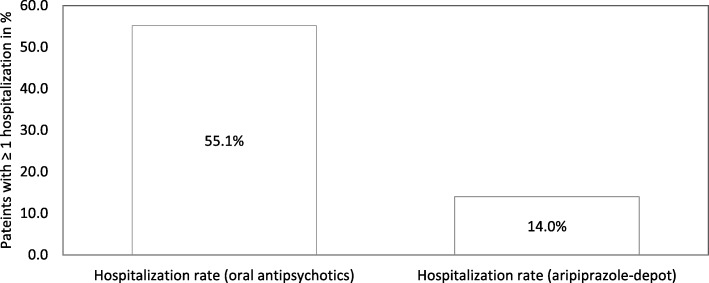
Total Hospitalization rates following the switch to aripiprazole-depot (Phase B) with the same patients treated with oral antipsychotics (Phase A). *P* value derived from Wilcoxon signed rank test

Psychiatric hospitalization rates (number of psychiatric hospitalizations ≥1) in patients who were treated with aripiprazole-depot (Fig. 2) were significantly lower compared to the retrospective hospitalization rates (months 1–6) when treated with oral antipsychotics.

The treatment with aripiprazole-depot also significantly reduced the mean number of hospitalizations (0.63 hospitalizations per patient during phase A vs. 0.16 hospitalizations per patient during phase B, *p* < 0.001) and the mean number of hospitalized days (27.39 (sd = 41.37) days in phase A vs. 5.56 (sd = 20.70) days in phase B, p < 0.001).

### Secondary efficacy end-points

The days of sick leave (resulting in a productivity loss) were examined for the employed subsample only. This restriction was justified by the irrelevance of days of sick leave in terms of contribution to the indirect costs of schizophrenia and reduced the sample size to *n* = 24 patients for this specific analysis. The mean number of days of sick leave during phase A was 40.06 compared to 15.80 days in phase B. This difference was not significant (*p* = 0.169).

The mean number of schizophrenia episodes was 2.58 (sd = 2.91) episodes per patient in the first six months during the treatment with oral antipsychotics, and 0.41 (sd = 0.76) for the second six month period with aripiprazole-depot treatment (*p* < 0.001).

The treatment change caused no significant reduction of the number of psychiatrist visits (10.57 to 7.73, *p* = 0.07) but instead there was a significant reduction of the mean stay in day-clinics and psychiatric institute ambulances (PIAs) (46.13 days (phase A) compared to 7.29 days (phase B), *p* < 0.01).

### Cost parameters

Cost savings were calculated from the perspective of the German statutory health insurance. The cost-relevant data for both treatment periods were derived from the cost variables in Table 2 and used in the BIA.

**Table 2:** Cost Parameters and Sources

Treatment	Source	Base rate
Productivity loss (days of sick leave)	Hannoveraner Konsens [18]	90 €
EBM^3^ Services	EBM (Einheitlicher Bewertungsmaßstab) [19]	**
GOÄ^4^ Services	GOÄ (Gebührenordnung für Ärzte) [20]	**
Days in day-clinics / PIA	BAG Psych. [21]	120 € - 360 €*
EBM Services during hospitalization	EBM [19]	**
GOÄ Services during hospitalization	GOÄ [20]	**
Hospital days	InEK PEPP / BBFW (GKV Spitzenverband) [22]	90 € - 385 €*
Emergency Services	EBM (Einheitlicher Bewertungsmaßstab) [19]	13.37 €
Emergency Physician	DGINA [23]	171 €
Non-drug-interventions	Various sources	**
Aripiprazole-depot injections	Rote Liste 2016	515.97 €
Medication	Rote Liste 2016	**
Medication (pre-existing conditions)	Rote Liste 2016	**

* Cost range considered in univariate sensitivity analyses, ** Costs vary by specific service / medicine

^3^Doctors’ Fee Scale within the Statutory Health Insurance Scheme

^4^Fee regulations for doctors within private Health Insurance Scheme

Both direct and indirect costs were considered. All the cost components were assessed in monetary units.

Table 3 shows per case costs of 9935.38€ for patients during oral-medication and 4557.56€ during aripiprazole-depot treatment. This suggests potential cost-savings of 5377.82€ per schizophrenia patient. 63,83% (1562.49€) of the total oral medication costs are caused by aripiprazole in its oral form. Even without consideration of these costs caused by oral aripiprazole prescriptions, the total costs per unit stay are significantly lower in the depot-treatment phase. The direct costs (without productivity losses) were also lower for the aripiprazole-depot study-period (9498.36 € vs. 4449.83 €).

**Table 3:** Costs per unit (*n* = 132)

Treatment	oral medication	aripiprazole-depot
Productivity loss (days of sick leave)	437.02 €	107.73 €
EBM Services	314.37 €	286.18 €
GOÄ Services	28.64 €	11.98 €
Days in day-clinics / PIA	121.21 €	63.64 €
EBM Services during hospitalization	78.36 €	26.02 €
GOÄ Services during hospitalization	4.67 €	1.29 €
Hospital days	5652.79 €	1288.04 €
Emergency Services	3.13 €	0.61 €
Emergency Physician	12.88 €	1.30 €
Non-drug-interventions	373.42 €	103.79 €
Aripiprazole-depot injections	0.00 €	2325.57 €
Medication	2447.80 € (885.31 €*)	103.35 €
Medication (pre-existing conditions)	461.07 €	238.07 €
Total	9935.38 € (8372.89 €*)	4557.56 €
Direct Costs (without productivity losses)	9498.36 €	4449.83 €

* without the costs for aripiprazole (oral medication)

Table 4 provides a breakdown of the total costs for the total study sample (*n* = 132) which were calculated under the assumption of a 100% substitution of schizophrenia patients with both treatment options. In this context, it should be noted that aripiprazole-depot is not a broad-based treatment option and is primarily prescribed for individuals with a history of non-adherence. Therefore, the projections in Table 4 must be interpreted as approximate estimates because a 100% substitution of aripiprazole-depot represents an unrealistic scenario.

**Table 4:** Total costs (*n* = 132)

Treatment	oral medication	aripiprazole-depot
Productivity loss (days of sick leave)	57,686.40 €	14,220.00 €
EBM Services	41.496,84 €	37,775.82 €
GOÄ Services	3.780,70 €	1581.62 €
Days in day-clinics / PIA	16,000.00 €	8400.00 €
EBM Services during hospitalization	10,344.07 €	3434.13 €
GOÄ Services during hospitalization	616.66 €	170.07 €
Hospital days	746,168.65 €	170,021.33 €
Emergency Services	413.67 €	80.22 €
Emergency Physician	1699.74 €	171.00 €
Non-drug-interventions	49,291.59 €	13,699.69 €
Aripiprazole-depot injections	0.00 €	306,975.32 €
Medication	323,109.37 € (116,860.96 €*)	13,642.75 €
Medication (pre-existing conditions)	60,861.86 €	31,425.42 €
Total	1,311,469.55 € (1,105,221.14 €*)	601,597.37 €
Direct Costs (without productivity losses)	1,253,783.15 €	587,377.37 €

* without the costs for aripiprazole (oral medication)

Treatment with aripiprazole-depot resulted in lower total costs compared to the oral medication treatment period (601,597.37€ vs 1,311,469.55€). This result remained similar also without consideration of the productivity losses (indirect costs) and distinctly supports the treatment with aripiprazole-depot. Hospitalizations and medications costs appear to be the main cost driving factors. During the treatment with aripiprazole-depot the reduced hospitalization-rates as well as the reduced number of hospitalized days resulted in significant cost savings compared to Phase A (170,021.33€ vs. 746,168.65€).

Other relevant cost driving factors were the productivity losses, the services (EBM / GOÄ) by the psychiatrists and the non-drug-interventions. Aripiprazole-depot remained the more cost-effective strategy for every single cost factor if medication costs were combined.^2^ Considering a prevalence rate between 0.8% and 0.9% [2] aripiprazole-depot treatment resulted in total costs between 2,989,756,603.05€ and 3,363,476,178.43€ compared to total costs between 6,517,606,265.43€ and 7,332,307,048.61€ (oral medication) for the total population of schizophrenia patients in Germany.^3^

### Sensitivity analyses

In order to examine the robustness of the results, a univariate sensitivity analysis was conducted for this BIA. For this purpose, one parameter is varied while all other parameters are kept constant. This way, the influence of specific variables on the outcome can be quantified. The resulting transition probabilities of the BIA were varied by the standard deviation (mean ± sd) of the cost-relevant variable. Cost uncertainty was varied by the cost-range for the cost-relevant variable.^4^

The following parameters were varied by using the subsequent values for each of both treatment options:

Probability of occurrence of:

- Productivity loss (days of sick leave)
  - ○ [oral antipsychotics: min = 10.44; max = 69.68; mean = 40.06; sd = 29.62]
  - ○ [aripiprazole-depot: min = 0.00; max = 40.47; mean = 15.80; sd = 24.67]
- Days in day-clinics / PIAs
  - ○ [oral antipsychotics: min = 0.66; max = 91.6; mean = 46.13; sd = 45.47]
  - ○ [aripiprazole-depot: min = 0.00; max = 22.31; mean = 7.29; sd = 15.02]
- Number of hospital days
  - ○ [oral antipsychotics: min = 0,00; max = 68.76; mean = 27.39; sd = 41.37]
  - ○ [aripiprazole-depot: min = 0.00; max = 26.26; mean = 5.56; sd = 20.70]
- Number of emergency physician treatments
  - ○ [oral antipsychotics: min = 0.00; max = 2.21; mean = 1.42; sd = 0.79]
  - ○ [aripiprazole-depot: min = 0.00; max = 3.00; mean = 2.00; sd = 1.00]
- Number of emergency service treatments
  - ○ [oral antipsychotics: min = 0.00; max = 4.91; mean = 2.38; sd = 2.53]
  - ○ [aripiprazole-depot: min = 0.00; max = 3.00; mean = 2.00; sd = 1.00]

Additionally costs were varied due to non-standardized costs for the following variables:

- Days in day-clinics / PIAs
  - ○ [both treatment options: min = 240.00 €; max = 720.00 €]
- Number of hospital days
  - ○ [both treatment options: min = 90.00 €; max = 385.00 €]

The results of the BIA remained robust to the modification of all seven uncertain cost-factors and probabilities during sensitivity analyses, with aripiprazole-depot being the more cost-effective strategy, with hospitalizations as the main cost-driving factor.

Costs for medication, psychiatrist services and non-drug-interventions were kept constant in the sensitivity analyses due to their characteristics (already being calculated as the sum of different medications, services and interventions), as no meaningful interpretable standard deviation could be calculated for the total costs.

The results of the sensitivity analysis are displayed in Table 5 (per case and total sample population) and Table 6 (extrapolation for the population of schizophrenia patients in Germany).

**Table 5:** Results of the univariate sensitivity analysis (Sample Population)

Treatment	Ø Per case costs (n = 132)		Ø Total costs (n = 132)	
	oral medication	aripiprazole-depot	oral medication	aripiprazole-depot
Base case analysis	9935.38 €	4557.56 €	1,311,469.55 €	601,597.37 €
Probability of occurence of productivity loss (days of sick leave)				
min	9612.25 €	4449.83 €	1,268,816.75 €	587,377.37 €
max	10,258.50 €	4725.76 €	1,354,122.35 €	623,800.37 €
Probability of occurence of days in day-clinics / PIA				
min	9894.16 €	4493.92 €	1,306,029.55 €	593,197.37 €
max	10,056.59 €	4621.19 €	1,327,469.55 €	609,997.37 €
Cost uncertainty of days in day-clinics / PIA				
min	9862.16 €	4493.92 €	1,301,805.55 €	593,197.37 €
max	10,250.53 €	4723.01 €	1,353,069.55 €	623,437.37 €
Probability of occurence / number of hospital days				
min	4282.58 €	3269.52 €	565,300.90 €	431,576.04 €
max	18,473.38 €	9352.96 €	2,438,486.61 €	1,234,590.43 €
Cost uncertainty of hospital days				
min	4282.58 €	3269.52 €	565,300.90 €	431,576.04 €
max	29,351.33 €	12,843.47 €	3,874,375.90 €	1,695,338.54 €
Probability of occurence of emergency physician				
min	9922.50 €	4556.26 €	1,309,769.81 €	601,426.37 €
max	9948.04 €	4561.82 €	1,313,141.33 €	602,160.27 €
Probability of occurence of emergency services				
min	9932.24 €	4556.95 €	1,311,055.89 €	601,517.15 €
max	10,037.63 €	4573.63 €	1,324,966.93 €	603,718.86 €

**Table 6:** Results of the univariate sensitivity analysis (Schizophrenia Population in Germany)

Treatment	Ø Total costs (*n* = 656,000)		Ø Total costs (*n* = 738,000)	
	oral medication	aripiprazole-depot	oral medication	aripiprazole-depot
Base case analysis	6,517,606,265.43 €	2,989,756.603.05 €	7,332,307,048.61 €	3,363,476,178.43 €
Probability of occurence of productivity loss (days of sick leave)				
min	6,305,634,774.52 €	2,919,087,512.14 €	7,093,839,121.34 €	3,283,973,451.16 €
max	6,729,577,756.34 €	3,100,098,784.87 €	7,570,774,975.88 €	3,487,611,132.98 €
Probability of occurence of days in day-clinics / PIA				
min	6,490,571,113.92 €	2,948,011,148.51 €	7,301,892,503.16 €	3,316,512,542.07 €
max	6,597,121,416.95 €	3,031,502,057.60 €	7,421,761,594.07 €	3,410,439,814.80 €
Cost uncertainty of days in day-clinics / PIA				
min	6,469,579,113.92 €	2,948,011,148.51 €	7,278,276,503.16 €	3,316,512,542.07 €
max	6,724,345,659.37 €	3,098,294,784.87 €	7,564,888,866.79 €	3,485,581,632.98 €
Probability of occurence of hospital days				
min	2,809,374,180.43 €	2,144,802,139.41 €	3,160,545,952.99 €	2,412,902,406.84 €
max	12,118,539,502.25€	2,296,772,366.69 €	13,633,356,940.03 €	2,583,868,912.52 €
Cost uncertainty of hospital days				
min	2,809,374,180.43 €	2,144,802,139.41 €	3,160,545,952.99 €	2,412,902,406.84 €
max	19,254,474,180.43 €	8,425,318,806.08 €	21,661,283,452.99 €	9,478,483,656.84 €
Probability of occurence of emergency physician treatments				
min	6,509,159,072.70 €	2,988,906,784.87 €	7,322,803,956.79 €	3,362,520,132.98 €
max	6,525,914,487.19 €	2,992,554,070.32 €	7,341,653,798.09 €	3,366,623,329.11 €
Probability of occurence of emergency services				
min	6,515,550,461.82 €	2,989,357,933.96 €	7,329,994,269.55 €	3,363,027,675.70 €
max	6,584,684,146.81 €	3,000,299,790.32 €	7,407,769,665.16 €	3,375,337,264.11 €

The base case analysis was already introduced in Table 3 and Table 4 and is used as a reference value for the variations in the sensitivity analysis. The results of the two therapy options remained robust to the removal of transitional probabilities and cost uncertainties. The probability of occurrence of days of sick leave (productivity loss) was varied by its own standard deviation and led to cost ranges of 9612.25€ to 10,258.50€ (oral medication) and 4449.83€ to 4725.76€ (aripiprazole-depot) per patient. The days in day clinics were varied in two different ways. The variation of the occurrence-probability by its own standard deviation led to a cost range of 9894.16€ to 10,056.59€ (oral medication) and 4493.92€ to 4621.19€ (aripiprazole-depot). Additionally the costs were varied for the day-clinic / PIA days because there is no fixed cost-value for these services. The variation of costs led to cost-ranges of 9862.16€ and 10,250.53€ (oral medication) and 4493.92€ to 4723.01€ (aripiprazole-depot).

The parameters hospital days and the associated costs of hospital days revealed the largest cost range and were identified as the parameters with the largest impact on parameter uncertainty. The variation of the occurrence-probability of the number of hospital days led to cost ranges of 4282.58€ to 18,473.38€ (oral medication) and 3269.52€ to 9352.96€ (aripiprazole-depot). Because of the fact that the hospitalization-costs are not standardized and differ significantly, costs for hospitalizations were varied as well. The cost-variation led to a cost range of 4282.58€ to 29,351.33€ for oral medication treatment and 3269.52€ to 12,843.47€ for aripiprazole-depot treatment. The parameters for emergency physician treatments / services achieved the lowest cost range. Therefore they achieved the smallest impact on parameter uncertainty. Table 5 shows these per case costs for the sample population (*n* = 132) while Table 6 extrapolates these costs under consideration of the estimated prevalence-rate of schizophrenia in Germany. The calculations for the German total population were made for both prevalence rates (0.8% / 0.9%). Table 7 shows the results under the assumption of a substitution of aripiprazole-depot between 4 and 12% for schizophrenia treatment [16].

**Table 7:** Results of the univariate sensitivity analysis (estimated market-share)

Treatment	Total costs (n = 656,000 * 0.04)		Total costs (n = 738,000 * 0.12)	
	oral medication	aripiprazole-depot	oral medication	aripiprazole-depot
Base case analysis	260.704.250,62 €	119.590.264,12 €	879.876.845,83 €	403.617.141,41 €
Probability of occurence of productivity loss				
min	252.225.390,98 €	116.763.500,49 €	851.260.694,56 €	394.076.814,14 €
max	269.183.110,25 €	124.003.951,39 €	908.492.997,11 €	418.513.335,96 €
Probability of occurence of days in day-clinics / PIA				
min	259.622.844,56 €	117.920.445,94 €	876.227.100,38 €	397.981.505,05 €
max	263.884.856,68 €	121.260.082,30 €	890.611.391,29 €	409.252.777,78 €
Cost uncertainty of days in day-clinics / PIA				
min	258.783.164,56 €	117.920.445,94 €	873.393.180,38 €	397.981.505,05 €
max	268.973.826,37 €	123.931.791,39 €	907.786.664,02 €	418.269.795,96 €
Probability of occurence of hospitalized days				
min	112.374.967,22 €	85.792.085,58 €	379.265.514,36 €	289.548.288,82 €
max	484.741.580,09 €	245.421.612,30 €	1.636.002.832,80 €	828.297.941,53 €
Cost uncertainty of hospitalized days				
min	112.374.967,22 €	85.792.085,58 €	379.265.514,36 €	289.548.288,82 €
max	770.178.967,22 €	337.012.752,24 €	2.599.354.014,36 €	1.137.418.038,82 €
Probability of occurence of emergency physician treatments				
min	260.366.362,91 €	119.556.271,39 €	878.736.474,82 €	403.502.415,96 €
max	261.036.579,49 €	119.702.162,81 €	880.998.455,77 €	403.994.799,49 €
Probability of occurence of emergency services				
min	260.622.018,47 €	119.574.317,36 €	879.599.312,35 €	403.563.321,08 €
max	263.387.365,87 €	120.011.991,61 €	888.932.359,82 €	405.040.471,69 €

## Discussion / limitations

The pre-post comparison study demonstrated significantly lower psychiatric hospitalization rates for patients switched from an oral antipsychotic therapy to aripiprazole-depot, as well as significantly less non-drug interventions and a reduced amount of productivity losses due to days of sick leave. The reduction of the hospitalization rates remained significant when being compared to a recent study and its reported hospitalization rate [11].

The BIA evaluated the costs and financial consequences of both schizophrenia treatments for the German health care system from the perspective of the German statutory health insurance. Treatment with oral-medication causes total costs of 6,517,606,265.43€, which are significantly higher than the total costs for treatment with aripiprazole-depot (2,989,756,603.05€). Considering the six-month observation period per treatment, these extrapolated costs for oral schizophrenia were significantly higher that the estimated annual schizophrenia costs in Germany (between 4,400,000,000€ and 9,200,000,000€ per year) [6].

Based on the study data, treatment with aripiprazole-depot is clearly more cost-effective than treatment with oral-medication for schizophrenia, which is mainly associated with less and shorter hospitalizations. These results support recent analyses which reported reduced health-care related costs for sustained release drugs compared to oral antipsychotics [17].

Regardless of the hospitalization-costs, aripiprazole-depot resulted in lower costs on all economical variables, including total drug-acquisition costs, which is an unexpected result considering the often reported greater initial acquisition costs associated with sustained release drugs [11]. These results can be put into perspective by the fact that 63,83% of the total medication costs in the oral treatment phase are caused by aripiprazole in its oral form.

Possible limitations of the pre-post comparison study are a potential selection bias of patients and psychiatrists due to missing randomization as well as reduced sample-sizes on variables that were analysed for the employed subsample only. There was no inclusion of a control group as the patients served as their own control. The pre-post study design additionally can be prone to biases because of time-changing context factors.

Additionally the sample sizes were comparatively low, especially with regard to the employed sub-population, as the study provides a pilot project to investigate the care situation of schizophrenia patients in Germany. The results should be tested and confirmed by future projects with significantly larger case numbers. These future research efforts should also focus more on the side effects of schizophrenia.

Some cost-variables were accessed by estimations. These estimations were documented and considered during sensitivity analyses, but despite that are inferior to empirically accessed cost data.

In that regard, the collection of additional economic data of the affected patients could complement the study data efficiently. Larger sample-sizes could help to enable inferential statistical procedures for subpopulations (like employed people only) and randomization in a prospective research design could lead to a reduction of selection bias. The present evaluation is also solely dealing with costs to the health insurance system and the patient himself and does not take into account other costs in terms of work productivity or time lost or burden to family members. The sensitivity-analysis which considered the estimated market-share of aripiprazole-depot was based on data for the UK [16]. Future analyses should take the German shares into account to reduce the bias generated due to the comparison of two different health-systems.

Another potential limitation is the fact that productivity losses were analyzed for the employed sample population only, which resulted in a reduced sub-population. It was not considered if the unemployment was caused by the schizophrenia. In this case, productivity losses and the associated costs would be systematically underestimated in the study results.

## Conclusion

The BIA contributes relevant data on the discussion of schizophrenia treatment in Germany that are in line with recent publications on the health-economic advantages of sustained release drugs like aripiprazole-depot compared to oral antipsychotic medication.

The results suggest that schizophrenia treatment with aripiprazole-depot instead of an oral antipsychotic treatment leads to significant cost reductions from the perspective of the German statutory health insurance.

The cost-saving potential is primarily explained by less and shorter hospitalizations combined with a smaller amount of productivity losses and a significant reduction of the mean stay in day-clinics and psychiatric institute ambulances (PIAs) for patients with aripiprazole-depot treatment.

## Abbreviations

BIA: Budget Impact Analysis
EBM: Einheitlicher Bewertungsmaßstab - Doctors’ Fee Scale within the Statutory Health Insurance Scheme
GOÄ: Gebührenordnung für Ärzte - Fee regulations for doctors within private Health Insurance Scheme
ICD-10: International Statistical Classification of Diseases and Related Health Problems
LAI: Long acting injectable
OA: Oral antipsychotics
PIA: Psychiatric institute ambulances
RCT: Randomized controlled trial
WHO: World Health Organization

## Footnotes

1 Calculation based on a total population of 82.800.000

2 Total medication costs were calculated by the sum of aripiprazole-depot injections (which were not used in Phase A), concomitant drugs and medications due to pre-existing conditions.

3 These costs were calculated for the duration of the observation period (6 months). For annual costs the amount has to be doubled.

4 Cost ranges for days in day-clinics / PIA and hospitalized days were used because of the absence of fixed costs on these variables and were accessed from the literature.
